# Glycemic index of some protein-free food products for individuals with non-dialysis-dependent chronic kidney disease

**DOI:** 10.1186/s12986-025-00990-5

**Published:** 2025-08-06

**Authors:** Alessandro Leone, Francesca Menichetti, Franca Criscuoli, Giovanni Fiorillo, Stefano Ravasenghi, Maria Cristina Casiraghi, Simona Bertoli

**Affiliations:** 1 International Center for the Assessment of Nutritional Status and the development of Dietary Intervention Strategies (ICANS-DIS), Department of Food, Environmental and Nutritional Sciences (DeFENS), University of Milan, Via Sandro Botticelli 21, Milan, 20133 Italy; 2 IRCCS Istituto Auxologico Italiano, Clinical Nutrition Unit, Department of Endocrine and Metabolic Medicine, Milan, 20100 Italy; 3 Department of Food, Environmental and Nutritional Sciences (DeFENS), University of Milan, Via Sandro Botticelli 21, Milan, 20133 Italy; 4 IRCCS Istituto Auxologico Italiano, Obesity Unit and Laboratory of Nutrition and Obesity Research, Department of Endocrine and Metabolic Diseases, Milan, 20145 Italy

**Keywords:** Chronic kidney disease, Glycemic index, Renal nutrition, Protein-free products

## Abstract

**Background:**

Chronic kidney disease (CKD) is a major public health issue and the third leading cause of death globally. In the conservative phase of CKD, a low-protein diet is recommended to slow disease progression, and protein-free products are commonly used in clinical nutrition for CKD. Since diabetes is highly prevalent in this population, it is crucial that such foods also have a low glycemic index (GI) to support glycemic control and reduce associated complications. This study aimed to assess the GI of selected commercial protein-free products.

**Methods:**

Twelve healthy volunteers (six males, six females; mean age 20.7 ± 0.8 years; BMI 22.6 ± 3.6 kg/m²) consumed four commonly available protein-free foods: sliced white bread, pasta, crackers, and cookies filled with vanilla cream (with sweeteners). The GI of each product was calculated according to ISO 2010 standards, using glucose as a reference. Each test meal provided 50 g of available carbohydrates.

**Results:**

GI values ranged from 48 for cookies filled with vanilla cream to 69 for crackers. Sliced white bread (GI 49.4) and cookies (GI 47.8) were classified as low-GI foods, while pasta (GI 68.2) and crackers (GI 69.2) fell within the medium-GI range.

**Conclusion:**

Several commercially available protein-free products exhibit low to moderate GI values, supporting their use in dietary management of patients with non-dialysis-dependent CKD and or at risk of diabetes. However, given the growing market of such products, further studies—including those on patients with CKD—are needed to expand the current evidence base.

## Introduction

Chronic Kidney Disease (CKD) is a pathological condition marked by a gradual yet progressive decline in kidney function [1]. Currently, CKD represents a significant global health issue and is the third fastest-growing cause of death worldwide [2]. According to recent estimates, CKD affects approximately 10% of the global population—over 800 million individuals—and 7–10% of the population in Italy [2, 3].

Without effective management, CKD can advance to end-stage renal disease (ESRD), the terminal stage of CKD in which kidney function is completely lost. At this point, renal replacement therapies, such as dialysis or kidney transplantation, become necessary [1]. Nutrition plays a pivotal role in decelerating progression to ESRD [4]. In the conservative phase of CKD (i.e., prior to dialysis), a low-protein diet may help slow the deterioration of kidney function, potentially extending the period of preserved renal function and improving clinical outcomes [5, 6]. To enhance dietary quality while limiting protein intake, it is essential to focus on high-quality protein sources and substitute traditional cereal-based foods—which typically contain moderate-quality proteins—with protein-free alternatives.

CKD is prevalent among the adult and elderly population, particularly in individuals with diabetes [7]. People with diabetes are significantly more susceptible to renal complications, including diabetic nephropathy, a specific form of kidney disease that substantially increases the risk of ESRD [8]. It is estimated that up to 50% of people with diabetes may develop diabetic nephropathy [9]. Additionally, research has highlighted a bidirectional relationship between kidney failure and diabetes [10]. The kidneys play a crucial role in regulating blood glucose levels by filtering the blood and selectively reabsorbing glucose as needed. When kidney function declines, glucose metabolism can be disrupted, promoting insulin resistance and, in turn, increasing the risk of diabetes. Therefore, in the conservative phase, the management of CKD involves not only the preservation of kidney function but also the prevention of diabetes in at-risk individuals, as well as the management of diabetes in those already affected [11]. A low-glycemic index (GI) diet can improve glycemic control in individuals with type 2 diabetes and may also help prevent weight gain, thereby reducing the risk of diabetes onset [12]. Consequently, it is important that protein-free products specifically developed for the nutritional management of non-dialysis-dependent (NDD) CKD also have a moderate-to-low glycemic index.

Therefore, we aim to evaluate the GI of different protein-free foods consumed by patients with NDD-CKD.

## Materials and methods

### Study design

The GI values of protein-free products were evaluated in a group of 12 healthy volunteers, comprising six males and six females, with a mean age of 20.7 ± 0.8 years and a mean BMI of 22.6 ± 3.6 kg/m². Volunteers were selected based on specific exclusion criteria, including diabetes, gastrointestinal disorders, use of medications affecting glucose tolerance, pregnancy, breastfeeding, and any known food allergies or intolerances. The Ethics Committee of the University of Milan approved the study procedures (protocol n. 21/20). All participants were fully informed about the study protocol and potential risks, and each provided written informed consent in line with the Declaration of Helsinki on human rights.

### Protein-free products

The protein-free products used in this study are representative of carbohydrate-based product types most commonly consumed by the Italian population. A total of 4 protein-free products were tested, including sliced white bread, pasta (penne), crackers, and a cookie filled with vanilla cream (with sweeteners). All products were manufactured and provided by FLAVIS, a brand of Dr. Schär AG/SPA, Italy. The list of ingredients used in the production of these products, along with their nutritional characteristics per 100 g of product, are detailed in Tables 1 and 2, respectively.

**Table 1:** Ingredients of low-protein products according to the producer labelling

Product	Ingredients
Pasta	maize starch, rice starch, thickener: cellulose; cane sugar syrup, chicory inulin, emulsifier: mono- and diglycerides of fatty acids; rice flour, safflower extract, color: beta-carotene
Sliced white bread	maize starch, water, rice starch, vegetable fiber (psyllium, chicory inulin, citrus, acacia), thickeners: cellulose, hydroxypropylmethyl cellulose, guar gum; rice syrup, yeast, sunflower oil, maize germ, dextrose, salt, acid: tartaric acid
Crackers	gluten free wheat starch, vegetable margarine [vegetable oils and fats (high oleic sunflower oil, shea butter) in varying proportions, water, salt, emulsifiers: mono- and diglycerides of fatty acids; lemon juice], maltodextrin, modified tapioca starch, maize flour, glucose syrup, thickener: cellulose; chicory inulin, thickener: guar gum; emulsifier: mono- and diacetyl tartaric acid esters of mono- and diglycerides of fatty acids; salt, acid: citric acid; raising agents: ammonium hydrogen carbonate, sodium hydrogen carbonate, natural flavoring
Cookies filled with vanilla cream (with sweeteners)	maize starch, vanilla cream 29% [sweetener: maltitol; coconut fat, potato starch, vegetable fiber (chicory inulin), emulsifier: sunflower lecithin; natural vanilla flavor], sweetener: maltitol; potato starch, coconut fat, gluten free wholegrain oat flour, maize flour, vegetable fibers (bamboo, chicory inulin, psyllium), sunflower oil, rice starch, apple extract, raising agents: ammonium hydrogen carbonate, sodium hydrogen carbonate; emulsifier: sunflower lecithin; natural vanilla flavor, thickener: xanthan gum

**Table 2:** Nutritional composition of protein-free products (per 100 g of product)

	Energy (kJ) Energy (kcal)	Protein (g)	Lipid (g)	Carbohydrates (g) of which sugars (g) of which polyalcol (g)	Fiber (g)	Serving (g)
Pasta	1485 351	0.5	1.1	81 3.2 0	7.3	61.7
Sliced white bread	937 223	0.8	2.4	43 4.8 0	13	116
Crackers	1831 436	0.8	13	75 2.7 0	7.7	66.6
Cookies filled with vanilla cream (with sweeteners)	1831 436	0.9	20	72 0.2 25.4	3.3	88.1

### Glycemic index protocol

The protocol used to measure and calculate the GI of the protein-free foods followed the guidelines outlined in the ISO 26642:2010 document [13]. On the test day, following overnight fasting, participants arrived at 08:00 at the International Center for the Assessment of Nutritional Status and Development of Dietary Intervention Strategies (ICANS-DIS), University of Milan. Upon arrival, two capillary whole blood samples were collected via finger prick (Accu-Chek Advantage System, Roche Diagnostics Limited, Lewes, UK), five minutes apart, in order to determine baseline blood glucose levels as the average of the two measurements. Subsequently, participants consumed either the test meal or the reference meal (glucose monohydrate), together with 500 mL of water. Additional capillary whole blood samples were collected at 15, 30, 45, 60, 90, and 120 min after meal consumption. Blood glucose levels were measured using an enzymatic method (Cobas Integra 400 Plus, Roche Diagnostics, Rotkreuz, Switzerland) on the same day as the test. Meal portions were determined based on the manufacturers’ nutritional information to provide 50 g of available carbohydrates. Since the cookies filled with vanilla cream contained maltitol, a polyol that is partially absorbed in the small intestine, 40% of the maltitol content in the food was considered for the calculation of 50 g of carbohydrates, as recommended in the guidelines outlined in the ISO 2010 document [13]. The protein-free pasta was cooked in 1 L of boiling water with 5 g of salt, with a cooking time of 11 min as indicated on the packaging. The products were administered following the computer-generated random order shown in Table 3.

**Table 3:** Administration order of protein-free products

id	Product 1	Product 2	Product 3	Product 4	Product 5	Product 6
1	bread	cracker	glucose	pasta	glucose	cookies
2	cracker	glucose	pasta	bread	cookies	glucose
3	glucose	bread	cookies	glucose	pasta	cracker
4	bread	pasta	cracker	glucose	glucose	cookies
5	cracker	glucose	cookies	pasta	glucose	bread
6	cookies	pasta	glucose	cracker	glucose	bread
7	glucose	bread	pasta	cracker	glucose	cookies
8	bread	cookies	glucose	glucose	pasta	cracker
9	cookies	glucose	bread	pasta	cracker	glucose
10	glucose	cracker	bread	cookies	pasta	glucose
11	pasta	glucose	cookies	bread	glucose	cracker
12	cookies	glucose	pasta	cracker	bread	glucose

### Statistical analysis

The results are expressed as mean ± standard error (SEM). The incremental areas under the curve (iAUC) were calculated geometrically by applying the trapezoid rule. GI was calculated as the percentage of the test product’s iAUC relative to that of glucose. Statistical analysis was performed using Excel as statistical software (Microsoft).

## Results

The main characteristics of the volunteers are presented in Table 4.

**Table 4:** Characteristics of the volunteers

	Males *N* = 6		Females *N* = 6		Total *N* = 12	
	mean	sd	mean	sd	mean	sd
Age (years)	20.8	0.8	20.5	0.8	20.7	0.8
BMI (kg/m^2^)	25.3	3.0	19.8	1.4	22.6	3.6

Data are presented as mean ± standard deviation (sd)

**Fig. 1:**
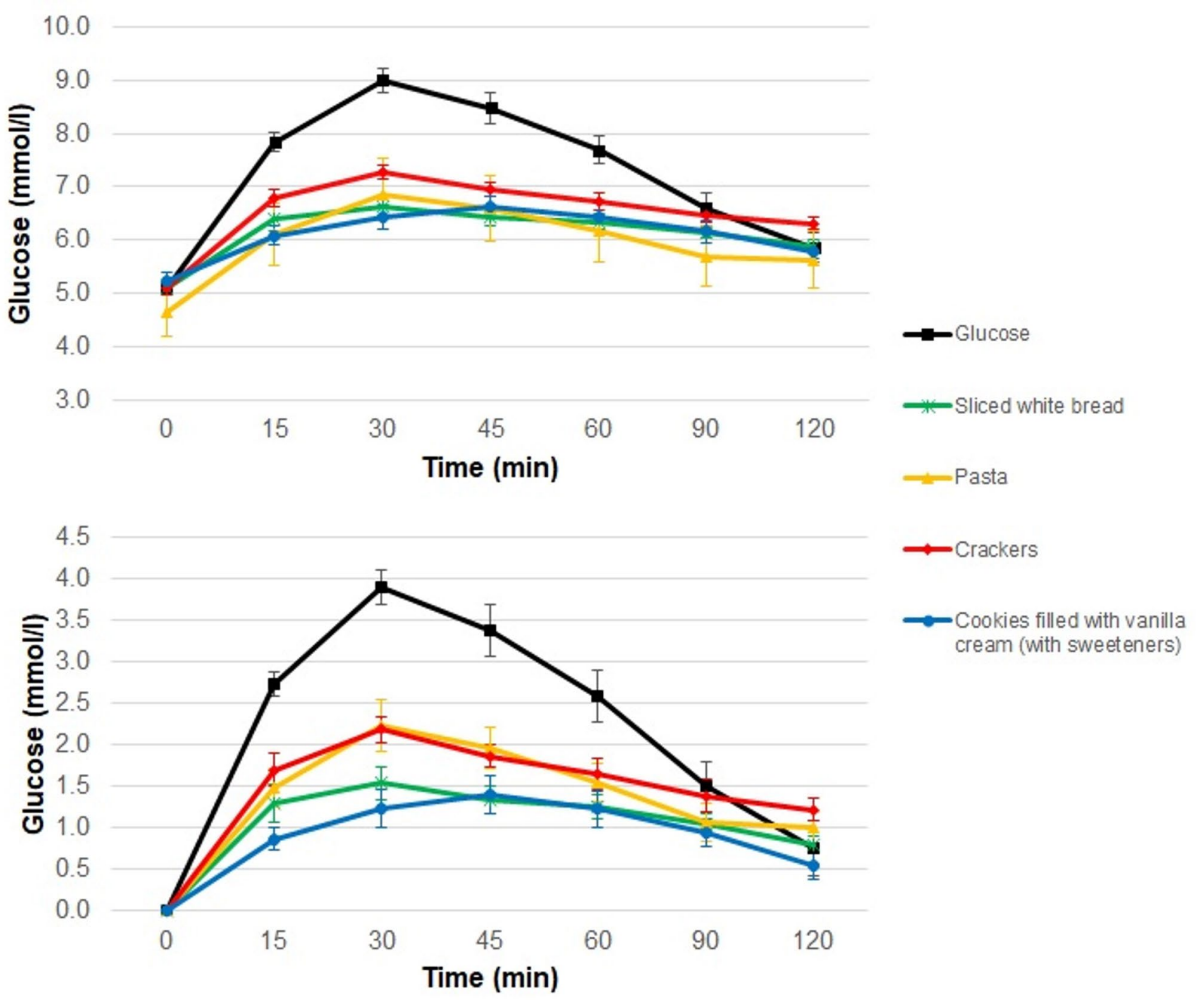
Glycemic responses obtained after administration of glucose and protein-free products. Glycemic responses (**A**) and incremental areas under the curve (**B**) obtained after administration of glucose and protein-free products. Values are means and standard errors

The glycemic curves obtained following the consumption of the protein-free products and glucose are shown in Fig. 1.

**Fig. 2:**
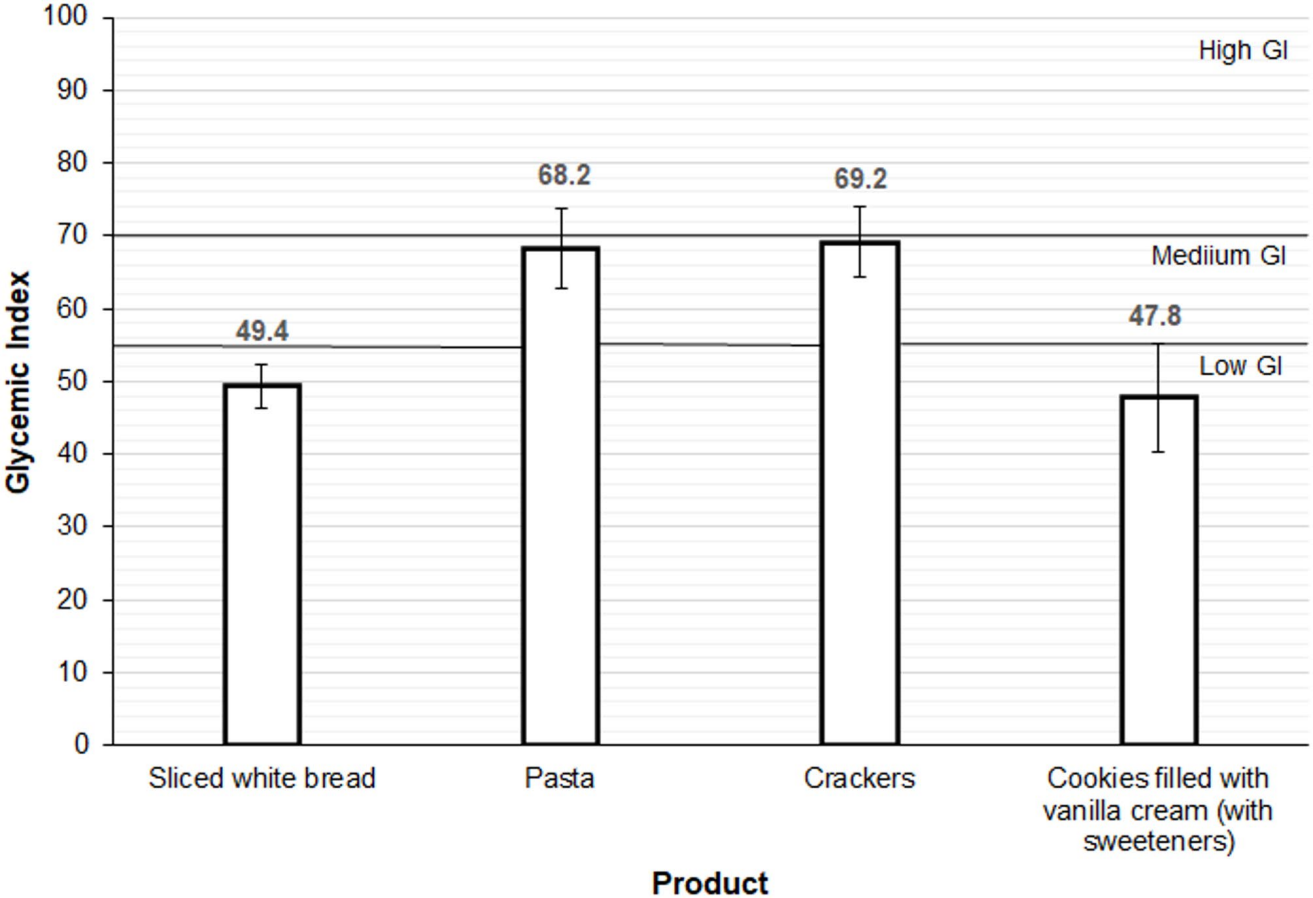
Glycemic index of protein-free produts. The graph shows the glycemic indices calculated as the percentage ratio of the incremental area under the curve of protein-free products to that of glucose. Values are means and standard errors

The GI values of protein-free products are shown in Fig. 2. Using the cut-off values reported in the literature, sliced white bread (GI: 49.4 SEM 3.0) and cookies filled with vanilla cream (GI: 47.8 SEM 7.5) had a low GI (< 55), while crackers (GI: 69.2 SEM 4.8) and pasta (GI: 68.2 SEM 5.5) had a medium GI (55–70).

## Discussion

The GI is a crucial tool for classifying foods based on their ability to influence postprandial blood glucose levels [14]. The consumption of foods with a low or moderate GI is particularly important for patients with NDD-CKD, a condition often associated with diabetes or an increased risk of developing it [15]. Managing CKD requires an integrated nutritional approach that addresses both slowing disease and achieving glycemic control to prevent or mitigate diabetic complications [1, 16].

The results of this study show that the four protein-free foods analyzed have GI values ranging from low to moderate, making them suitable for inclusion in the diet of patients with NDD-CKD. Specifically, sliced white bread and cookies filled with vanilla cream (with sweeteners) fall into the low GI category, while pasta and crackers exhibit moderate GI values. Notably, none of the tested products exhibited a high GI, indicating their potential to support effective blood glucose management.

A comparison between the GI values obtained in this study and those of traditional food products reported in the literature reveals some important differences. These differences may be attributed to variations in nutritional composition and the nature of the ingredients used. Protein-free sliced white bread showed a significantly lower GI compared to its conventional counterpart, which is typically classified as a medium-GI food [17]. Notably, the bread analyzed in this study had a higher fiber content (13%) than traditional bread. Additionally, specific types of fiber, such as guar gum, psyllium, inulin, and hydroxypropyl methylcellulose, used in the production of protein-free sliced white bread, are known to delay gastric emptying and reduce the postprandial glycemic response. It has been previously demonstrated that white bread prepared with 5% guar gum reduced its GI by 41% [18]. Another study reported that consuming white bread with 15 g of psyllium decreased the postprandial glycemic response by approximately 40% [19]. Furthermore, an additional study [20] found that adding 12% inulin reduced the GI of bread by 32%. Regarding the cookies filled with vanilla cream, they exhibited a low GI, consistent with values reported in the literature for traditional biscuits [17]. Although almost entirely devoid of protein, the analyzed cookies had a lipid content similar to that of conventional biscuits. The presence of lipids is known to delay gastric emptying, thereby reducing the postprandial glycemic response. Moreover, as observed for bread, the inclusion of chicory inulin and psyllium may have contributed to the product’s low GI. Regarding pasta, a moderate GI was observed, higher than the values reported for traditional pasta [17] but comparable to those of certain gluten-free pasta varieties [21]. The higher GI, compared to its traditional counterpart, can be attributed to its low protein content and increased starch availability. Unlike Durum wheat pasta, protein-free pasta is produced using purified starches (in our case, maize and rice starches). This likely facilitates starch gelatinization during cooking, making the starch in protein-free pasta more accessible to digestive enzymes. Finally, the crackers exhibited a moderate GI, comparable to that of traditional crackers. Their high lipid content—similar to that of conventional crackers—along with a higher fibre content, including soluble fibre such as inulin, may have mitigated the absence of protein and contributed to slowing gastric emptying.

From a clinical perspective, these findings are highly significant. The analyzed protein-free foods not only fulfill the essential requirement of limiting protein intake to slow the progression and manage signs, symptoms and complications of NDD-CKD but also contribute to glycemic control, which is critical for reducing the risk of CKD progression, preventing type 2 diabetes, and managing associated complications. A recent systematic review reported a strong association between greater glycemic variability and the onset and progression of CKD [22]. Other studies have further demonstrated that higher glycemic variability increases the risk of progression to ESRD [23, 24, 25]. Poor glycemic control is particularly associated with a faster decline in glomerular filtration rate (GFR) [26]. On the other hand, low-glycemic index (GI) foods have been shown to minimize blood glucose fluctuations [27, 28], thereby reducing glycemic variability [29, 30]. Although no studies to date have directly evaluated the effects of low-GI diets on renal function in patients with CKD, evidence from a randomized controlled trial suggests that, in adults with overweight and obese, diets emphasizing low-GI foods are associated with improved GFR compared to high-GI diets [31]. The adoption of low-GI foods may offer additional benefits to patients with NDD-CKD by reducing the risk of developing diabetes, a frequent comorbidity in this population. A recent meta-analysis of prospective studies identified a linear relationship between dietary GI and diabetes risk, with a 32% increase in risk for every 10-unit rise in GI [12]. Another critical benefit of consuming low-GI foods is the potential reduction in cardiovascular risk. Patients with diabetic kidney disease are at high risk for cardiovascular events and mortality. A comprehensive meta-analysis of 27 randomized controlled trials involving 1,617 diabetic participants reported that, compared to high-GI diets, low-GI diets not only significantly improve blood glucose control but also enhance lipid profiles, reduce systolic blood pressure, and decrease inflammation—all factors closely tied to cardiovascular risk [32]. A recent prospective study reported that a diet characterized by high-GI foods was associated with a 21% increased risk of major cardiovascular events or death, even among individuals without pre-existing cardiovascular disease [33]. The incorporation of lower GI foods into the dietary management of NDD-CKD may therefore provide a dual benefit: optimizing glycemic control and reducing cardiovascular risks, thereby enhancing clinical outcomes for patients.

## Conclusion

This study demonstrates that several commercially available protein-free products exhibit low to moderate glycemic index values. These findings are important for the nutritional management of individuals with non-dialysis-dependent chronic kidney disease, for whom protein restriction and glycemic control are both essential. However, the results obtained from the analysis of only four products are insufficient to draw definitive conclusions about the glycemic index values of protein-free products, given the vast array of options currently available on the market. Indeed, due to the rapid increase in the number of protein-free formulations, further research is strongly recommended to support dietary management of patients with non-dialysis-dependent chronic kidney disease. Future studies should also include patients with chronic kidney disease to establish a comprehensive database of glycemic index values for protein-free products.

## Data Availability

The dataset analyzed during the current study is available from the corresponding author on reasonable request.

## Abbreviations

CKD: Chronic kidney disease
ESRD: End-stage renal disease
NDD: Non-dialysis-dependent
GI: Glycemic Index
iAUC: Incremental areas under the curve
GFR: Glomerular filtration rate
